# Does Value Stream Mapping affect the structure, process, and outcome quality in care facilities? A systematic review

**DOI:** 10.1186/s13643-017-0563-y

**Published:** 2017-08-24

**Authors:** Marina Nowak, Holger Pfaff, Ute Karbach

**Affiliations:** Institute of Medical Sociology, Health Services Research and Rehabilitation Science (IMVR), Faculty of Human Sciences, Faculty of Medicine, University of Cologne, Eupener Strasse 129, 50933 Cologne, Germany

**Keywords:** Value Stream Mapping, Lean management, Quality improvement, Organizational development, Systematic review

## Abstract

**Background:**

Quality improvement within health and social care facilities is needed and has to be evidence-based and patient-centered. Value Stream Mapping, a method of Lean management, aims to increase the patients’ value and quality of care by a visualization and quantification of the care process. The aim of this research is to examine the effectiveness of Value Stream Mapping on structure, process, and outcome quality in care facilities.

**Methods:**

A systematic review is conducted. PubMed, EBSCOhost, including Business Source Complete, Academic Search Complete, PSYCInfo, PSYNDX, SocINDEX with Full Text, Web of Knowledge, and EMBASE ScienceDirect are searched in February 2016. All peer-reviewed papers evaluating Value Stream Mapping and published in English or German from January 2000 are included. For data synthesis, all study results are categorized into Donabedian’s model of structure, process, and outcome quality. To assess and interpret the effectiveness of Value Stream Mapping, the frequencies of the results statistically examined are considered.

**Results:**

Of the 903 articles retrieved, 22 studies fulfill the inclusion criteria. Of these, 11 studies are used to answer the research question. Value Stream Mapping has positive effects on the time dimension of process and outcome quality. It seems to reduce non-value-added time (e.g., waiting time) and length of stay. All study designs are before and after studies without control, and methodologically sophisticated studies are missing.

**Conclusions:**

For a final conclusion about Value Stream Mapping’s effectiveness, more research with improved methodology is needed. Despite this lack of evidence, Value Stream Mapping has the potential to improve quality of care on the time dimension. The contextual influence has to be investigated to make conclusions about the relationship between different quality domains when applying Value Stream Mapping. However, for using this review’s conclusion, the limitation of including heterogeneous and potentially biased results has to be considered.

**Electronic supplementary material:**

The online version of this article (doi:10.1186/s13643-017-0563-y) contains supplementary material, which is available to authorized users.

## Background

Health and social care organizations face challenges as a result of the aging population, as well as the increasing number of patients with chronic and multiple diseases. Together with the increasing shortage of specialists and the decline in employment, potentially resulting from demographic trends in high-income countries, the quality of care is at risk [1, 2]. Quality is further compromised by growing demands on employees arising from increasingly complex technologies or organizational deficits [3]. These deficits, such as interface problems, hygiene shortcomings, or diagnostic errors in service providing institutions, are often rooted in problems related to the system of care provision rather than the individuals involved [3, 4]. Therefore, improving quality within the health and social care sector requires changes which should in the best case avoid further investment and rather focus on organizational restructuring [5].

Lean management, developed for the automotive industry in Japan, summarizes principles, methods, and procedures to structure organizations and their processes effectively and efficiently. It aims to increase value for the customer and minimize waste. Wastes are processes and activities which bind resources but produce no value [6]. Lean management refers to a way of thinking which enables the recognition of value and the separation and organization of value-added and non-value-added time and process steps. The work flow of organizations changes following this value, producing only the outcome defined by the customer. For some time, this approach has been considered as a possibility for organizational development in health care organizations [7, 8]. Thereby, the patient becomes the customer, and the care processes improve based on the patient’s perspective, adding value, and decreasing waste for him [8].

Value Stream Mapping (VSM) is a method of Lean management. It has the potential to improve complex workflows by addressing the customer’s needs through visualization and quantification. VSM is used within the context of Lean and Lean Six Sigma interventions, whereby it seems to be an important part of these methods [8, 9]. In particular, in the United Stated of America (USA), the actors in health care already use VSM to minimize waste such as waiting times or to avoid unnecessary process steps (e.g., distances) [10].

Applying this method of process improvement in the context of care organizations is challenging due to the dynamics involved and flexibility required in the process flow within personalized services compared to volume production within industrial applications [11, 12]. However, there are reviews of Lean and Six Sigma in general, applied in surgical services [13] and emergency departments [14] demonstrating a positive effect. Contrary, another review does not support effects of Lean management within various health care settings [15]. A further review is not able to make any final conclusion about its effectiveness in health care organizations [16], probably resulting from a lack of quality in evaluation studies [16, 17] or very different applications of the Lean concept [18]. Reviews of VSM within the health care sector highlight its importance within Lean management yet made no statement about its effectiveness [8, 9, 19]. Therefore, an assessment of VSM’s actual effect on the quality of care is essential.

Consequently, this review aims to investigate whether VSM is suitable for use in care facilities. More specifically, the project at hand examines the effectiveness of VSM on the structure, process, and outcome quality of health and social care settings with respect to patient-centeredness.

### Conceptual framework

For the purpose of properly assessing and measuring quality in care organizations, more conceptual understanding of quality in care is required. Health science research employs Donabedian’s model of structure, process, and outcome quality (SPO framework) which conceptualizes quality as a three dimensional construct [20–22]. Structure quality describes the environment of the care organization, such as equipment or pathways, but also qualifications of staff or administrative structures. Process quality defines the way of performing health or social services (e.g., treatment delays or use of procedure). Finally, outcome quality is the direct change in the health status of the patient and includes the patients’ satisfaction as well as the entire care process [21]. Breaking down quality into these three domains helps to categorize the outcomes of the studies considered in this review. This facilitates the assessment of the single studies’ contributions and ultimately allows the synthesizing of their results.

### Value Stream Mapping

The VSM method includes the visualization of complex work flows, quantification of the resources needed (e.g., staff, materials, time), and restructuring of the work flows into an improved version with focus on the patient’s needs [23]. Therefore, VSM aims to reduce unnecessary process steps and time. These aspects are non-value adding, as they create no value for the patient [23]. Simultaneously, process steps and time which improve quality of the process for patients, e.g., face-to-face contact with the physician [24], are aimed to be increased. These are services which patients would be willing to pay for [10] and are called value adding [23].

**Fig. 1:**
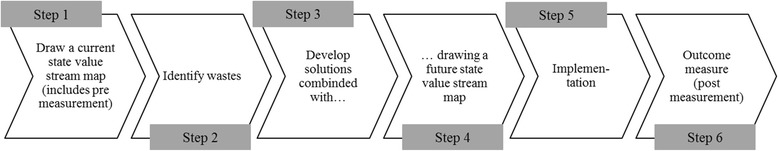
Six steps of Value Stream Mapping

VSM can be described in six phases [23, 25] shown in Fig. 1. In the first phase of the VSM method, a current state value stream map of a process is developed, including a pre measurement. The second phase follows with identifying wastes based on this map. After that, in the third phase, solution approaches for improvements of the process are developed, being converted into a future state value stream map in the fourth phase. Subsequently, in the fifth phase, an implementation of the new process is conducted, finishing with an outcome measure (post measurement) as the sixth and last phase.

## Methods

For the purpose of answering the research question, a systematic literature review is carried out. Reporting is done according to the “Preferred Reporting Items for Systematic Reviews and Meta﻿-Analyses” (PRISMA) scheme [26] (see Additional file 1 for the PRISMA checklist).

### Registration

The project is registered in a project database of German Network Health Services Research (http://www.versorgungsforschung-deutschland.de/show.php?pid=2705) [27]. The detailed review protocol is added as Additional file 2, but it is not registered at the international prospective register of systematic reviews (PROSPERO).

### Information sources

A sensitive search strategy is developed to identify all relevant studies. Studies are identified by searching electronic databases and applying the search to PubMed, EBSCOhost, including Business Source Complete, Academic Search Complete, PSYCInfo, PSYNDX, SocINDEX with Full Text, as well as Web of Knowledge and EMBASE ScienceDirect. Dates of coverage are January 1, 2000 to February 15, 2016, whereby the latter is the date last searched.

### Search

The search strategy consists of two components. The first component represents “Value Stream Mapping” and its synonyms; those are combined with the Boolean operator “OR.” The second component embodies all names and synonyms for “care organizations” which are also combined via “OR.” Finally, these two components are combined with “AND” to only include studies meeting both criteria. The search strategy is applied to the different databases (see Additional file 3) to fulfill special requirements or keywords (e.g., MeSH terms for PubMed). If possible, the search of the components is restricted to the title and abstract.

### Eligibility criteria and study selection

Title screening, abstract screening, and full-text screening are conducted by two reviewers independently to assess whether the studies fulfill the inclusion criteria. Disagreements are resolved through discussion.

Studies evaluating VSM or a concept named differently but using the same technique are included. VSM has to be applied to any type of care facility, such as hospitals or social care institutions. For studies to be considered, it is required that the intervention included at least six phases by Rother and Shook [25] and Jimmerson [23] described above (see Fig. 1). This guarantees homogeneity of the interventions employed. Different implementation methods or methods to analyze the time of the process within the VSM application are allowed. All objectively reported outcomes are included.

All peer-reviewed papers published in English or German from January 2000—because one of the initial descriptions in industrial contexts of VSM was in this time [28]—to February 15, 2016 are included to ensure quality assurance and completeness of coverage. The study design has to be quantitative. Secondary literature, such as commentaries, editorials, opinions or perspectives and dissertations, posters, literature research, reviews, and qualitative research, as well as VSM applications in other than care settings, are excluded to increase the homogeneity of studies.

### Data collection process

The data is extracted from the articles by the reviewer MN. The reviewer UK checks the extracted data. Discussion is used to solve disagreements. Microsoft Excel 2016 is used to manage the references and to collect all extracted data in one spreadsheet.

### Data items

The data items, authors, year of publication, study design, intervention details (procedure, involved persons, contribution, duration, and further interventions), setting (institution, department, and country), aim, sample size, and all reported outcomes, changed and unchanged, are extracted when given. The procedure of intervention is used to check for the six phases of VSM. It is assumed that all outcomes can be mapped on to the SPO framework and by clustering they are simplified. Furthermore, the reported outcomes are simplified by reducing on whether the study authors examine and report results statistically in comparison to no statistical analyses. When available, means, standard deviances, 95% confidence intervals, and significance levels are extracted.

### Risk of bias in individual studies

After study selection, quality assessment is conducted in three ways. First, the study design’s level of evidence is assessed as proposed by the Oxford Centre for Evidence-Based Medicine (OCEBM) [29]. Second, risk of bias is assessed based on the recommendations of the Cochrane Collaboration, thus by study design [30]. These results do not influence the data synthesis. Third, a further quality assessment is based on whether the studies examine their results statistically (e.g., inferential statistics) and present significance tests. Only these are used to assess the effectiveness of VSM.

### Summary measures and synthesis of results

Due to an expected heterogeneity in methodological approaches and outcomes, no meta-analysis is conducted. Additionally, it is decided not to develop a forest plot of the studies’ results because of too much variation (e.g., time measurements). Therefore, the synthesis and analysis of the results has a descriptive character. To synthesize the results, all studies are mapped on to the SPO framework based on literature [20, 21]. In addition, in an inductive step, results are mapped on to subcategories for process, structure, and outcome quality (e.g., non-value-added or value-added time for process quality). This procedure ensures a finer grained synthesis and does not overly generalize.

In order to assess and interpret the effectiveness of VSM, only studies fulfilling the third quality assessment are considered. If the data allows, means are listed and 95% confidence intervals are extracted or calculated out of standard deviations. The frequency of the outcomes in each quality dimension is determined.

### Risk of bias across studies

Because of the expected high levels of heterogeneity between studies, assessing the risk of bias across studies is infeasible (e.g., variation in setting or outcome measure). However, in order to minimize the risk of selective reporting within studies, all given results are included.

## Results

**Fig. 2:**
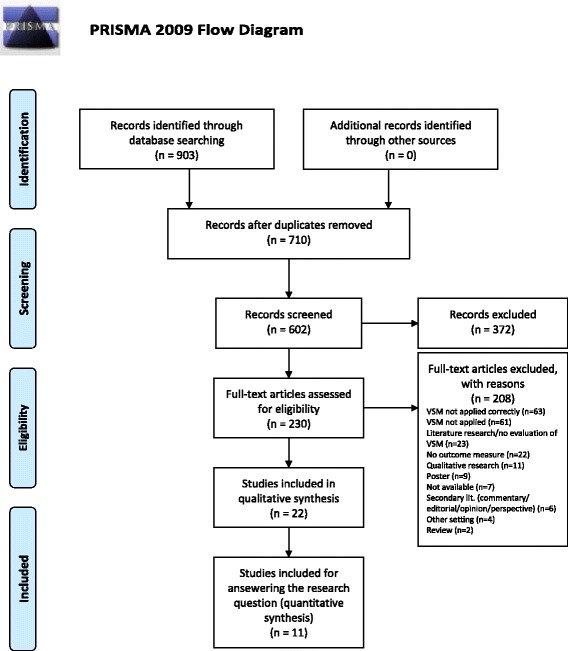
Flow chart of the search and selection process

Figure 2 shows the process of study selection. The search strategy identifies 602 peer-reviewed articles (after removal of duplicates). After title screening, 329 articles proceed to abstract screening. This further reduces the number to 230 relevant for full-text screening, whereby 7 are not available. Finally, 22 studies fulfill the inclusion criteria for the qualitative synthesis.

The study’s authors, country, setting, year of publication, name of intervention, procedure of intervention, stated aim, study design, sample size, as well as all reported results, changed and unchanged, are extracted. Involved persons and contribution are not extracted because of little information in articles. Information about procedure of intervention is only used to check whether the interventions follow the six phases of VSM but is not used for analysis. Additional file 4 gives an overview of the included studies and data items.

Of all 22 studies, the studies that most often applied VSM are carried out in the USA (50%). Three of the 22 studies are conducted in Canada, two in the UK, and one each in Brazil, China, India, Spain, Ireland, and Sweden.

The chosen approach includes studies from various settings within care facilities. Thereby, the application areas, as well as the accuracy in describing the setting, differ between the studies. VSM is applied to the emergency department in four of the 22 studies. Five studies are conducted in surgical services or operating theaters directly. In five studies, the authors carry VSM out to improve administration processes, whereby one of these studies is also applied in a surgical service department. Pharmacy or medication operations are examined in three studies. Two further studies are conducted within outpatient units. The setting of the remaining four studies cannot be categorized (e.g., inpatient rehabilitation unit or complex chronic care management).

All of the studies have a before and after design without control. Three studies further include a mixed methods approach. The research is mostly descriptive, even when using a before and after design. Following the OCEBM, all studies are graded at Level IV [29], when assuming the before and after design without control being at least as evident as case series.

Within the second quality assessment, the risk of bias with critical appraisal checklists following the study design, as suggested by the Cochrane Collaboration [30], is planned. The Cochrane Collaboration does not provide any checklist for before and after studies without control. They suggest treating the results with caution [30] as internal validity is threatened. To differentiate between the studies and get insight into their internal validity, the methodological index for non-randomized studies (MINORS) [31] is used retrospectively. As suggested by Zeng and colleagues [32], it is an excellent tool for the assessment of non-randomized interventional studies.

Overall, none of the studies met all the criteria to avoid risk of bias. Only three studies [24, 33, 34] reach a score of 10 or more, whereby the total score is 16. In average, the studies only reach a score of 5.59, ranging from 1 to 13 (see Additional file 4). The background within the introduction is described in all studies, and mostly the objectives are described adequately. However, the authors generally do not include a theoretical context in form of a contextual or logical model. A study protocol is only reported once [33]. The likelihood of biased assessment is given for most of the studies. One single study addresses potential sources of bias by including an observer effect period. This is to control for unintended effects of reactive measurement, describing the effect of changing one’s actions because of being observed and evaluated. Finally, only some studies include prospective calculations of the study size, describing it adequately only once.

All results of the 22 studies are mapped on to the SPO framework (see Table 1). Therefore, subcategories are developed inductively. These are volume of patients (e.g., changed incoming calls for appointment, number of patients per month), staff reallocation (e.g., changed utilization of staff), education of staff (e.g., changed educational experience of surgical residents), and room change (e.g., changed walking distances) for structure quality. Within process quality, the subcategories are non-value-added time (e.g., changed waiting times or time from initial call until appointment was scheduled), non-value-added process steps (e.g., changed number of patients’ appointments canceled without being seen or number of errors), value-added time (e.g., changed face-to-face contact between physician and patient), and staff satisfaction (e.g., changed morale of staff). Finally, mortality/infection, length of stay (e.g., changed total processing time or turnaround time), costs (e.g., changed weekly expenses), and patient satisfaction are subcategories for outcome quality.

Half of the studies (11 studies) examine results statistically and thereby fulfill the third quality assessment criteria. These studies are used to answer the research question, and the minimum quality standard ensures the reliability of this review’s results.

**Table 1:** Results of included studies mapped on to quality domains and subcategories

Quality domains and subcategories	Structure quality				Process quality				Outcome quality			
Included studies	Volume of patients	Reallocation of staff	Education of staff	Room change	Non-value-added time	Non-value-added process steps	Value-added time	Staff satisfaction	Length of stay	Costs	Mortality/infections	Patient satisfaction
Arbune et al. [57]				○	○				○			
Bhat et al. [58]		●			●				●			
Chiodo et al. [59]					○		○					
Collar et al. [33]			x		● x			●	●			
Ford et al. [41]					● x				x		x	
Godinho Filho et al. [60]	○				○	○			○	○	○	
Kim et al. [61]					○	○			○			
L’Hommedieu and Kappeler [34]	○					●				●		
Martin et al. [62]				○	○	○		○	○			
Mazur et al. [63]										○		
Mazzocato et al. [24]	●				●				●			
McDermott et al. [37]					●		● x		●			
McJoynt et al. [40]									●			
Michael et al. [38]	○					● x			●			
Ng et al. [64]	○				○	○			○			○
Reznick et al. [36]	●				●	●						
Rico and Jagwani [65]						○	○					
Sampalli et al. [66]					○							
Skeldon et al. [35]	x				● x	○	●		●			
Wojtys et al. [67]					○	○						○
Yousri et al. [39]					x	x			x		●	
Zhu et al. [68]	○				○							

● Improvement/change: significant result

x No improvement/change: no significant result

○ Observed improvement/change: no statistical analysis

### Effect of Value Stream Mapping on the quality of care

In this section, all outcomes of the 11 studies that examine results statistically and provide significance levels (see Additional file 4) are used to answer the research question concerning the effect of VSM on the quality of care based on Donabedian’s quality domains.

Outcomes of the five studies are categorized into structure quality, whereas only three studies produced statistically significant results. The volume of patients or material/products is measured in three studies, e.g., decreased clinic volume [35] or increased number of overall patients per month [36]. However, the results are ambiguous such that no clear conclusion concerning VSM’s effectiveness on structure quality can be drawn.

Ten of the 11 studies include outcome measures for process quality. Most of them (nine studies) use at least two outcome measures for process quality. Non-value-added time is measured in eight studies. Thereby, VSM achieve a statistically significant improvement in seven of these studies. For example, a reduction of waiting time from initial call to consult and from consult to operation [36], or the time from arrival to the time the physician is seen [37] are observed. Non-value-added process steps were measured statistically in four studies. Here, the number of errors for the doses within pharmacy operations [34] as well as the number of patients who canceled appointments without being seen [36] is reduced by a statistically significant magnitude. However, the authors of two articles find ambiguous [38] or negative [39] results. Two studies measure value-added time such as face-to-face contact with a physician or nurse [35, 37], producing varying results. Staff satisfaction is measured only once such that no effect can be assumed. Although some aspects of process quality are not affected by VSM, the time aspects prove to be responsive to VSM interventions.

The outcome quality is measured in ten of the 11 studies. However, only length of stay is measured in more than two studies. Seven studies out of nine find a statistically significant reduction of length of stay, e.g., a reduction of the total patient journey time [37], a reduced total processing time from receipt to result [38], or a reduced turnaround time for internally authored protocols [40]. In contrast, Yousri and colleagues [39] and Ford and colleagues [41] are not able to confirm a statistically significant reduction of length of stay. Nevertheless, it is concluded that the time dimension, in this case length of stay, is affected by VSM.

## Discussion

Overall, the results show that there are no studies with high methodological quality to make a final conclusion about VSM’s effectiveness in health and social care organizations. All Lean applications described in the studies produce at least one positive effect through VSM, including no negative effect on the quality of care. A common but no less important challenge of all systematic reviews is handling publication and reporting bias [14, 18]. In the review at hand, only one of the included studies finds more non-statistically significant results than otherwise [39]. No study finds a negative effect of the VSM method. Risk of bias of individual studies is high in most studies, but their results are still included. Further, risk of bias across studies is not feasible to assess because of the studies’ heterogeneity. Therefore, the results have to be interpreted with caution.

Nevertheless, the results indicate that VSM, as proposed by Rother and Shook [25] and Jimmerson [23], has a positive effect on process and outcome quality in health care facilities. Process quality is mainly affected in terms of a reduction of non-value-added time, thus confirming the expectations derived from different (e.g., automotive) industrial applications [42]. This aspect is patient-centered because e.g., long waiting times have an effect on the satisfaction of patients [43, 44]. However, an improvement of value-added time, such as face-to-face contact with physicians, can influence this effect on patient satisfaction [45] implying the importance of a balanced improvement of the quality categories.

The change in outcome quality is represented by a reduction of length of stay. A correlation between the reduced non-value-added time and length of stay and causation can be suggested. For an evaluation of VSM, it is important to observe length of stay or total processing time in order to evaluate whether the reduction is actually creating value. For example, a time reduction of subprocesses with total processing time remaining constant could result in either an increase of non-value-added time in other subprocesses or in more value-added time (e.g., medical conversation with nurse). Thereby, only the latter would produce patient-oriented value. On the other hand, a reduction of non-value-added time (e.g., waiting time) can also result in a reduced total processing time instead of an increase in value-added time. This effect is also an improvement but would show other results. Consequently, VSM can help to increase value for the patient on the time dimension in two ways. Firstly, reducing non-value-added time can liberate time resources within and for the same process (e.g., for a reallocation to value-added process steps). Secondly, by reducing the total processing time, VSM can unlock other resources such as reduced costs for care facilities, thus allowing the use of existing resources (e.g., finances, staff) in other areas.

The mapping of outcomes on to the SPO framework dimensions was done by the first author, verified by the last, and based on literature. This process still involves subjectivity. It was assumed that all outcomes could be mapped on to the dimensions, which implies a forced choice. Some results are interconnected as described above (e.g., time for subprocesses and total length of stay), which complicated the clustering process. Further, dividing the quality domains into institutional and individual patient levels would bring the possibility of making even finer grained syntheses because the current subcategories do not differentiate between these aspects, potentially missing important information.

The result of this review, that VSM can affect the time aspects of health care, holds in various settings within health care, such as administrative or acute care processes. That is given for Lean management in general e.g., [13, 14]. VSM emerged out of the automobile industry and continues to produce positive effects [46, 47] especially when continuing to develop [48]. In comparison, the effects found in health care settings are generally weaker. That is because these settings differ from the industrial application [49] as they are more dynamic, less predictable, and require more flexibility [11, 12]. A main orientation of health services research is on the patient and what is improving the patient’s value [5], which can be fulfilled by orienting the process around the patient [50]. But a particular challenge when applying process improvement methods such as VSM in the care context is the organizational culture, the willingness to change in general, and the attitude of the staff [24, 50, 51]. These aspects influence the implementation success of Lean management and its methods [52]. Therefore, in advance of implementing VSM to care settings, it is essential to inform and train the staff about the Lean concept and its goals, thereby eliminating fears and misunderstandings, so that the improvement process is not impaired [52, 53].

To investigate the effects of VSM, as a complex intervention, it is required to evaluate the methods within their context. For the evaluation of complex interventions, it is important to include several aspects and fulfill the requirements to be able to generalize the results [54, 55]. Process evaluation, including a logical model, improves the understanding of complexity and dynamics between context and intervention [56]. Including a mixed methods design would further increase the understanding of the mode of action of VSM on the quality [55]. Only three of the studies utilized mixed methods, whereby the qualitative results have not been included in this review. No study included contextual or logical models. The development of those models, based on tested results, is therefore required.

For a comprehensive understanding of VSM and its effect in care facilities, the aspects of quality influenced directly by the application of VSM have to be measured and analyzed. Based on the results, these are primarily changes in time. Not all authors of the studies collected and analyzed the total processing time or length of stay e.g., [42]. Most of the studies simply measured and reported outcomes, which were available for measurement, potentially missing important variables (e.g., costs, patient satisfaction, success of implementation, willingness to change). A study protocol, which increases transparency of the measured variables, was only reported once [33]. Further, in two studies, length of stay did not change significantly, but in one of those, the mortality decreased [39] and in the other [41] time for subprocesses changed but health outcomes were unaffected. A comprehensive contextual model and a process evaluation would support the understanding of the connection between VSM and quality of care (e.g., mortality) as well as avoid omission in collecting data on important variables. As mentioned above, a mixed methods approach can expand the knowledge of this mode of action. For example, Mazzocato and colleagues [24] found that staff emphasized the reduction of “individual working ambiguity” (p. 10) or a “connection between caregivers who were dependent on one another” (p. 10). Other studies, such as Hung and colleagues [52] analyzed the factors influencing the staff’s acceptance of Lean redesign within primary care, whereby implementation success is affected by the style of implementation and employee engagement. This review is limited because qualitative study designs and qualitative results of mixed methods designs are excluded. A systematic review and potentially more research including these aspects have to be conducted to analyze any causation.

The articles used in this review all include the six steps of VSM proposed by Rother and Shook [25] and Jimmerson [23]. Thereby, the comparability and homogeneity of the intervention is ensured even when sometimes employed differently. Nevertheless, the studies still show heterogeneity. Firstly, the name of the intervention used, mentioned in the title or abstract, differs between the studies, most of them using “Lean,” “Value Stream Mapping,” or “Lean Six Sigma” (see Additional file 3). Secondly, VSM is sometimes combined with methods such as time series analysis or a fishbone root cause analysis. This second aspect, in particular, could affect the results. To overcome the difficulty that VSM can be used as a start and end control of a process change as well as an independent method [9], this review only includes studies of the latter. However, only a few articles met the inclusion criteria. The potential is given that the search strategy, even though developed with high sensitivity, missed essential research articles.

A further limitation is given through the different settings. It is likely that the application of VSM within emergency departments differs from its application within administrative processes such as protocol development. Further, none of the included studies concentrate on social care settings. A systematic review of the effectiveness of VSM on specific areas of health or social care departments can increase the validity. In future, more studies of high methodological quality have to be conducted.

Carrying out a systematic review without randomized controlled trials threatens its validity. Therefore, the strength of evidence is limited. The results are potentially biased as described above and should be improved, particularly in methodological terms. However, randomized controlled trials for evaluating complex interventions are not always feasible, as realistic and more pragmatic settings are often favorable. Well-designed observational studies can integrate these advances [55]. For determining the effectiveness of VSM, studies examining results statistically are used, disregarding the magnitude of observed effects. Further research can overcome this limitation by including homogeneous study designs and recalculating and controlling the results. Vest and Gamm [17] stress the need for more high qualitative evaluations of Lean management and Six Sigma in the health care context. This systematic review concludes that the same is still needed for VSM. Nevertheless, it is the best evidence available and complies with the expectations of the theory. Therefore, the results pave the way for future research to further specify its effectiveness in health and social care settings.

## Conclusions

This review concludes that a final and evidence-based evaluation of VSM in health and social care organizations cannot yet be made. However, it is assumed that an application of VSM has a positive effect on the process and outcome quality of health care organizations on a time dimension. More specifically, it seems to be able to reduce non-value-added time such as waiting times and length of stay, increasing value for the patient.

Further research with high methodological quality is needed with respect to the study design. This should especially be performed with a focus on statistical analyses and within logical and contextual models to fulfill the guidelines concerning evaluations of complex interventions.

## Additional files

Additional file 1: Includes the PRISMA checklist with the page numbers referring to the review manuscript: “Does Value Stream Mapping affect the structure, process, and outcome quality in care facilities? A systematic review”. (DOC 64 kb)

Additional file 2: Shows the review protocol in the format provided by PROSPERO. The protocol includes the information out of the planning process and the adaptations made during the review process. (DOCX 27 kb)

Additional file 3: Includes a table with the search strategies adapted to different electronic databases. (XLSX 11 kb)

Additional file 4: Includes an overview of the included studies. The table shows information about included studies (reference number), country, setting, name of intervention, stated aim, study design, sample size, risk of bias, score MINORS [31], results (clustered into structure, process, and outcome quality), summary statistic of significant results (mean with 95% confidence interval, if available or computable). (XLSX 19 kb)

## Abbreviations

MINORS: Methodological index for non-randomized studies
OCEBM: Oxford Centre for Evidence-Based Medicine
PRISMA scheme: Preferred Reporting Items for Systematic Reviews and Met﻿a-Analyses scheme
PROSPER﻿O: International prospective register of systematic revie﻿ws﻿
SPO framework: Structure, process, and outcome quality framework
VSM: Value Stream Mapping
