# Supplementary material for: Does Value Stream Mapping affect the structure, process, and outcome quality in care facilities? A systematic review
Source: Syst Rev. 2017 Aug 24;6:170. doi: 10.1186/s13643-017-0563-y (PMC5571664; doi:10.1186/s13643-017-0563-y)
Supplement: Supplementary file 2 — Shows the review protocol in the format provided by PROSPERO. The protocol includes the information out of the planning process and the adaptations made during the review process. (DOCX 27 kb) [file 13643_2017_563_MOESM2_ESM.docx]

**Additional file 2**

**Review protocol.**

1. **Review title ***

Value Stream Mapping in care organizations – an evidence-based method of organizational development?

Adapted to: Does Value Stream Mapping affect the structure, process and outcome quality in care facilities? A systematic review

1. **Original language title**

Not applicable

1. **Anticipated or actual start date ***

01 October 2015

1. **Anticipated completion date ***

01 April 2016

Adapted to: December 2016 (completion of manuscript and first submission)

1. **Stage of review at time of this submission ***

The review has not yet started [ ]

Preliminary searches Started

Piloting of the study selection process Started

Formal screening of search results against eligibility criteria Started

Data extraction

Risk of bias (quality) assessment

Data analysis

Provide any other relevant information about the stage of the review here (e.g. Funded proposal,

protocol not yet finalised).

Protocol not yet finalized

1. **Named contact ***

Marina Nowak

1. **Named contact email ***

[Marina.Nowak@uk-koeln.de](mailto:Marina.Nowak@uk-koeln.de)

1. **Named contact address**

Eupener Str. 129, 50933 Köln, Germany

1. **Named contact phone number**

+49 221 478 97108

1. **Organisational affiliation of the review ***

Institute for Medical Sociology, Health Services Research and Rehabilitation Science, Faculty of Human Sciences and Medicine, University of Cologne

[http://www.imvr.de/](http://www.imvr.uni-koeln.de/)

1. **Review team members’ and their organisational affiliations**

Dr. Ute Karbach, Institute for Medical Sociology, Health Services Research and Rehabilitation Science, Faculty of Human Sciences and Medicine, University of Cologne

Prof. Dr. Holger Pfaff, Institute for Medical Sociology, Health Services Research and Rehabilitation Science, Faculty of Human Sciences and Medicine, University of Cologne

1. **Funding sources/sponsors ***

The review is funded by the Institute for Medical Sociology, Health Services Research and Rehabilitation Science, Faculty of Human Sciences and Medicine, University of Cologne

1. **Conflicts of interest ***

All authors declare that they have no conflicts of interest.

1. **Collaborators**

Not applicable

1. **Review Question(s) ***

Does Value Stream Mapping have an effect on the quality of care organizations?

Adapted to: Is Value Stream Mapping suitable for use in care facilities?
Does Value Stream Mapping has an effect on the structure, process and outcome quality of health and social care settings with respect to patient-centeredness?

1. **Searches ***

We will search the following electronic bibliographic databases: PubMed, EBSCOhost (Business Source Complete, Academic Search Complete, PsycInfo, PSYNDEX, SocINDEX with Full Text), Web of Science, EMBASE ScienceDirect

The search strategy will include terms describing the intervention “Value Stream Mapping”. It has to be sensitive and therefore includes terms relating to “Value Stream Mapping”, e.g. “Lean” in combination with “analysis of flow”. The search terms will be adapted for each database in combination with database-specific filters, when available.

All studies in German or English will be included. All studies published between 2000 (01 January) and the date latest searched will be included.

Adapted to: Latest date searched: 15 February 2016

1. **URL to search strategy**

Adapted to: The search strategy was developed during the search process. Additional file 3 shows the full strategy adapted to the electronic databases

1. **Condition or domain being studied ***

Value Stream Mapping/Analysis, effects in care settings

1. **Participants/population ***

Inclusion: All types of care facilities as hospitals or social care institutions, Exclusion: Industries or any other domain

1. **Intervention(s), exposure(s) ***

Value Stream Mapping is a Lean Management technique used within health and social care settings. It is a special type of flow chart which uses symbols to describe the current and future situation of a process. A process can describe patient flow as well as material or information flow or a combination out of it. It should include a current and a future map which can also be recorded within a table. If the described method is comparable (same mechanisms) but does not use the terms “Value Stream Map”, “future map” or “current map” it will also be included. Furthermore, at best representatives of all occupation groups involved in the process have to participate, but at least some different occupation groups (>1) should be involved (except the process is just for one occupation group) as well as a facilitator (or comparable) for designing the current and future map.

Adapted to: The Intervention has to include at least six phases by Rother and Shook [25] and Jimmerson [23] (see Figure 1):

“In the first phase of the VSM method a current state value stream map of a process is developed. The second phase follows with identifying wastes based on this map. After that, in the third phase, solution approaches for improvements of the process are developed being converted into a future state value stream map in the fourth phase. Subsequently, in the fifth phase an implementation of the new process is conducted, finishing with an outcome measure as the sixth and last phase.”

1. **Comparator(s)/control ***

Not applicable

1. Types of study to be included initially *

All types of studies relevant to the research question will be included. We are looking for experimental and observational studies. Case reports can also be included. We will not include editorial reports, book chapters or conference talks, posters, comments or discussion, letters, publications without references and qualitative studies.

Adapted to: Articles published in not peer-reviewed journals are excluded.

1. **Context ***

Studies of all countries will be included if published in English or German. All kinds of social and care facilities will be included.

1. **Primary outcome(s) ***

All objectively reported measures will be included. We will include outcomes concerning e.g. time, efficiency, cost, patient satisfaction, staff satisfaction, practice changes, sustainability of the practice changes, through-put, complications/waste, aspects of teamwork, professional outcomes, adverse events, work loss reduction, access, hospitalization rates, length of stay, chronic disease rates and mortality.

1. **Secondary outcomes ***

We will not divide into primary and secondary outcomes.

1. **Data extraction (selection and coding)**

Titles and abstracts of studies retrieved using the search strategy will be screened independently by two review authors to find appropriate studies fulfilling the criteria. The full text of these studies will be retrieved and independently screened. If still fulfilling the criteria the quality assessment is also done by the two review authors. Any disagreement over the eligibility will be resolved through discussion.

We will use a form to extract the important data of the studies to assess the quality and conduct the evidence synthesis. The data extraction will be done by two review authors independently. Extracted information will include (in process):

First author, year of publication, study design, intervention details (exact procedure, involved persons, contribution, duration, further interventions), setting (institution, department and country), aim, outcomes.

Adapted to: Quality assessment is done by the first author and verified by the last author.

Extracted data items are: authors, year of publication, study design, intervention details (procedure, involved persons, contribution, duration and further interventions), setting (institution, department and country), aim, sample size and all reported outcomes, changed and unchanged are extracted when given

1. **Risk of bias (quality) assessment ***

The assessment will be done by two review authors independently. Articles published in not peer-reviewed journals will be excluded. Further risk of bias assessment will be done but will not imply exclusion of the studies:

Were statistical tests used to determine significant improvement?

Was the study free of any bias which could have affected the outcome?

Adapted to:

Criteria of articles published in not peer-reviewed journals is switched to exclusion/inclusion criteria.

Quality assessment is done in three steps:

1. The study design’s level of evidence is assessed as proposed by the Oxford Centre for Evidence-Based Medicine (OCEBM)
2. Risk of bias is assessed based on the recommendations of the Cochrane Collaboration, thus by study design.

These results do not influence the data synthesis.

1. Quality assessment is based on whether the studies examined their results statistically (e.g. inferential statistics) and presented significance tests. Only these are used to assess the effectiveness of VSM.
2. Strategy for data synthesis *

The results will be presented descriptively. For data synthesis the outcomes will be clustered into Donabedian’s categories of structure, process and outcome quality and interpretation concerning effectiveness and practicability is conducted based on these clusters.

Adapted to: To allow a finer grained synthesis, results are mapped on to subcategories for process, structure and outcome quality (e.g. non-value-added or value-added time for process quality)

1. **Analysis of subgroups or subsets ***

Analysis of subgroups is not anticipated and planned

1. **Type of review**

Systematic review

1. **Language**

English, German

1. **Country**

No restrictions

1. **Other registration details**

Project registration at a German project database: <http://www.versorgungsforschung-deutschland.de/show.php?pid=2705>

1. **Reference and/or URL for published protocol**
2. **Dissemination plans**

A paper will be submitted to a leading journal in this field.

1. **Keywords**

Systematic review; Value Stream Mapping; Lean Management; Analysis of flow; Health and social care settings

Adapted to: Value Stream Mapping, Lean management, quality improvement, organizational development, systematic review

1. **Details of any existing review of the same topic by the same authors**

Not applicable

1. **Review status**

Ongoing

Adapted to: finished

1. **Additional information**

None

1. **Link to publication of final report**
